# Frequency of dementia among elderly admitted to a Geriatrics Inpatients Sector of a Brazilian public hospital

**DOI:** 10.1590/1980-57642018dn12-010005

**Published:** 2018

**Authors:** Rafael Thomazi, Liciana Vaz de Arruda Silveira, Paulo José Fortes Villas Boas, Alessandro Ferrari Jacinto

**Affiliations:** 1 MD. Internal Medicine Department, Botucatu Medical School - São Paulo State University (UNESP), SP, Brazil; 2 PhD. Associate Professor, Instituto de Biociências Botucatu, São Paulo State University (UNESP), SP, Brazil; 3 MD, PhD. Associate Professor, Internal Medicine Department, Botucatu Medical School - São Paulo State University (UNESP), SP, Brazil

**Keywords:** aged, inpatient, dementia, idoso, hospitalização, demência

## Abstract

Patients with dementia are commonly admitted to inpatient sectors. The aim of this study was to describe the frequency of dementia among elderly inpatients admitted to the Geriatrics Sector of a Brazilian Tertiary University Hospital, and to identify associations between dementia and clinical and sociodemographic factors.

**Methods:**

All patients admitted to the Geriatrics Sector of a public Brazilian university-hospital from March 1^st^ 2014 to January 31^st^ 2015 were assessed by geriatricians. The patients were divided into groups "with or without diagnosis of dementia". Univariate analysis was performed between these two groups using the Chi-Square Test, Student's t-test or the Mann-Whitney Test.

**Results:**

One hundred and three elderly inpatients, with a mean age of 82 (±7.9) years, were assessed. Overall, 74.7% had low educational level (<4 years), 66% used polypharmacy, 57.2% developed delirium during hospitalization and 59% were totally dependent for basic activities of daily living. The diagnosis of dementia was observed in 59 (57%) subjects.

**Conclusion:**

The frequency of dementia was high among the elderly inpatients evaluated. The association between dementia and certain clinical conditions, such as incontinence, delirium and use of psychoactive drugs, was in line with the medical literature.

## RESUMO

Pacientes com demência são comumente admitidos em setores de internação. O objetivo deste estudo foi descrever a frequência de demência em pacientes idosos internados em uma enfermaria de geriatria de um hospital universitário terciário brasileiro e identificar associações entre demência e fatores clínicos e sociodemográficos.

**Métodos:**

Todos os pacientes internados na enfermaria de geriatria de um hospital universitário público brasileiro no período de 1 de março de 2014 a 31 de janeiro de 2015 foram avaliados por geriatras. Os pacientes foram divididos em dois grupos: com ou sem diagnóstico prévio de demência. Análise univariada foi utilizada na comparação dos resultados dos dois grupos por meio do Teste do Qui-Quadrado, teste t-Student e o teste de Mann-Whitnney.

**Resultados:**

Foram avaliados 103 pacientes idosos, com idade média de 82 (±7,9) anos; 74,7% apresentaram escolaridade baixa (<4 anos), 66% usavam polifarmácia, 57,2% desenvolveram delirium durante a hospitalização e 59% eram totalmente dependentes das atividades básicas da vida diária. O diagnóstico de demência foi observado em 59 indivíduos (57%).

**Conclusão:**

A frequência de demência foi alta entre pacientes idosos avaliados. A associação entre demência e certas condições clínicas, como a incontinência, o delírio e o uso de drogas psicoativas, está em consonância com a literatura médica.

The world's elderly population continues to grow at an unprecedented rate. Currently, 8.5 percent of people worldwide (617 million) are aged 65 and over, a figure set to rise to 17% by 2050.^1^ Population ageing, along with improvements in health care and socioeconomic conditions, is paradoxically contributing to the increased prevalence of dementia.^2^ Worldwide, dementia rates range from 5% to 7%.^3^ In Latin America, the rate is approximately 7%,^4^ while in Brazil, ranges from 5.1% to as high as 19.0%.^5^^,^ 6

Worldwide, 47 million are people living with dementia and this number is projected to increase to more than 131 million by 2050. The total estimated worldwide cost of dementia is US$ 818 billion.^7^

Dementia appears to be common in older patients admitted to acute hospitals and is associated with a range of adverse outcomes during hospitalization, including *delirium* , longer hospital stays, falls and increased mortality.^8^^-^ 14

The aim of this study was to describe the frequency of dementia among elderly inpatients admitted to the Geriatrics Sector of a Brazilian tertiary university hospital, and to identify associations between dementia and clinical and sociodemographic factors.

## METHODS

A cross-sectional study assessing all patients admitted to the Geriatrics Sector of the "Hospital das Clínicas de Botucatu" (HCB) between March 1^(st)^ 2014 and January 31^(st)^ 2015 was conducted. The data collected was used to create a database containing sociodemographic, pharmacological and clinical aspects of the inpatients. The diagnosis of dementia was established prior to hospitalization by other doctors and corroborated by geriatricians. The criteria for the diagnosis of dementia published by Frota et al. were used.^15^ Polypharmacy was defined as the continuous use of four or more drugs. Functional status was determined by the Katz Index. For the evaluation of delirium, the Confusion Assessment Method (CAM) was used.^16^

There were no exclusion criteria.

The Geriatrics Sector has eight beds run by a medical team comprising two residents of the Residency Program in Geriatrics of the "Faculdade de Medicina de Botucatu - UNESP" supervised by three Associate Professors of Geriatrics. The HCB is one of the largest public institutions in the State of São Paulo and part of the Brazilian Unified Health System. It is located in the city of Botucatu. The hospital serves 68 cities in an area with a population of two million.

The project was submitted for approval to the Local Research Ethics Committee (65573417.6.0000.5411).

### Statistical analysis

A descriptive analysis of the whole sample was performed. The sample was divided into two groups: "dementia" and "non-dementia". Chi-Square Test and Student t-test were used for comparisons between the two groups. The level of statistical significance adopted was 0.05​.

### RESULTS

One hundred and three elderly inpatients, with a mean age of 82 (± 7.9) years, were assessed. A descriptive analysis of sociodemographic, functional capacity, pharmacological and clinical factors is given in Tables 1 and 2. Regarding sociodemographics and functional capacity, 58.2% of the patients were male, 78% had low educational level, 21% lived in long-term care institutions, and 59% were totally dependent for basic activities of daily living on the Katz scale.^17^ In terms of pharmacological medical aspects, 66% were in use of polypharmacy, 33% antidepressants, 26% neuroleptics, 24% benzodiazepines and 20% acetylcholinesterase inhibitors. For clinical aspects, 64% had hypertension, 57,2% dementia, 57.3% *delirium* , 42% depression and 46% urinary incontinence.

**Table 1:** Sociodemographic, functional capacity and pharmacological aspects of the sample.

		N	%
Gender	Male	60	58.2
	Female	43	41.7
Marital status	Married	30	29
	Single	5	5
	Divorced	8	8
	Widowed	60	58
Race	Black and other	17	16.4
	White	86	83.6
Dwelling	Botucatu	89	86
	Other	24	14
Education (years)	> 4	26	25.2
	≤4	77	74.7
Polypharmacy (Four or more medications used)	Yes	68	66
	No	35	34
Psychotropic use	Antidepressants	35	34
	Neuroleptics	27	26
	Benzodiazepines	25	24
	Anticholinesterases	21	20
Dependency for activities of daily living	Independence	13	13
	Partial dependence	29	28
	Total dependence	61	59
Feeding	Tube feeding	23	33
	Oral feeding	80	77

**Table 2:** Clinical aspects of the sample.

	N	%
Hypertension	66	64
Dementia	59	57.2
Delirium	59	57.2
Pneumonia	44	42.7
ICU	16	15.5
Depression	37	35
Diabetes	34	33
Dyslipidemia	31	30
Falls in the last year	31	30
Urinary incontinence	44	42
Stroke	29	28
Heart Failure	30	29
Pressure lesion (admission)	44	42.7
Pressure injury (acquired)	35	34
CKD	17	16
Osteoarthrosis	48	46
Osteoporosis	26	25
Previous fractures	26	25
COPD	10	9
Deaths	35	34

UTI: urinary tract infection; CKD: chronic kidney disease; COPD: chronic obstructive pulmonary disease.

Palliative care support, including end-of-life care, was provided to 16% of inpatients, with a total of 34% deaths among all patients admitted to the geriatrics ward during the study period.

There was a significant statistically difference between the "dementia" and "non-dementia" groups in body mass index (BMI) and serum albumin level (SAL). Both BMI and SAL were higher in the "non-dementia" group, as shown in Table 3.

**Table 3:** Comparison of "dementia" and "non-dementia" groups for body mass index and serum albumin level.

	Dementia Mean ( ± SD)	Non-dementia Mean ( ± SD)	p
BMI	18.7 (16.8;21.4)	22.3 ( ± 4.9)	0.01*
SAL	2.45 (2.1;3.0)^#^	2.9 ( ± 0.6)	0.01**

* Student's *t*-test;

** Mann-Whitney Test;

# Median (Interquartile Range); SD: standard deviation; IQR: interquartile range; BMI: body mass index; SAL: serum albumin level.

Comparison of "dementia" versus "non-dementia" groups revealed that patients with dementia used more psychotropic drugs, had more urinary incontinence, were more dependent for basic activities of daily living and developed more *delirium* during hospitalization. All other variables showing a statistically significant difference between the two groups are depicted in Table 4.

**Table 4:** Variables with statistically significant association between "dementia" and "non dementia" groups.

		Dementia			Non-dementia		P*
		(N)	%		(N)	%	
Psychoactive drug	Yes	45	76.2		19	43.1	0.001
	No	14	23.7		25	56.8	
Neuroleptic	Yes	24	40.6		3	7.1	<0.001
	No	35	59.3		39	92.8	
HF	Yes	11	18.6		19	43.1	0.007
	No	48	81.3		25	56.8	
COPD	Yes	2	3.3		8	18.1	0.01
	No	57	96.6		36	81.8	
Incontinence	Yes	40	67.7		4	9	<0.001
	No	19	32.2		40	90.9	
DBADL	Yes	48	81.3		13	29.5	<0.001
	No	11	18.6		31	70.4	
Delirium	Yes	41	69.4		18	40.9	0.004
	No	18	30.5		26	59	
Pressure Lesion (admission)	Yes	33	55.9		11	25	0.002
	No	26	44		33	75	
Pressure lesion (acquired)	Yes	27	45.7		8	18.1	0.003
	No	32	54.2		36	81.8	
Caregiver	Yes	24	40.6		8	18.1	0.01
	No	35	59.3		36	81.8	
LTCI	Yes	17	28.8		5	11.3	0.03
	No	42	71.1		39	88.6	
Bedridden	Yes	45	76.2		14	31.8	<0.001
	No	14	23.7		30	68.1	

* Chi-Square test; HF: heart failure; COPD: chronic obstructive pulmonary disease; DBADL: dependency for basic activities of daily living; LTCI: long-term care institution.

## DISCUSSION

The frequency of dementia at the Geriatrics Sector of the HCB was 57.2%, a higher rate than that reported in other studies conducted mostly in Internal Medicine Inpatients Sectors. The frequency of dementia in elderly inpatients^8^ has been reported as between 20 and 30%. A higher frequency of dementia was expected in a Geriatric Inpatient Sector than in Internal Medicine Inpatient Sectors. One of the studies showing a higher frequency of dementia (63%) was conducted at a Geriatrics Inpatient Sector in an older, mainly female population.^18^ In the present study, the frequency of dementia was also higher than the rate of approximately 7% found among community-dwellers,^3^ suggesting more frequent hospitalization among the elderly with dementia. Patients with dementia had poorer functional ability than patients without dementia, a ﬁnding also reported recently elsewhere.^19^ This group was more likely to have *delirium* at admission and to develop *delirium* during admission than patients without dementia. Our ﬁndings conﬁrm previous reports regarding the importance of dementia as a risk factor for *delirium* during hospitalization.^20^ This population also exhibits high rates of incontinence, use of psychotropic drugs, longer hospital stays and higher rates of new institutionalization.^21^ To summarize, it is not dementia itself that leads an elderly patient to be admitted to hospital, but the biologically fragile state that results from dementia and the associated comorbidities.^22^

In a prospective study involving a cohort of very old acutely ill geriatric inpatients, higher levels of comorbidity and poor functional status were more predictive than dementia for adverse hospitalization outcomes.^23^ This highlights the importance of carrying out a more in-depth evaluation of hospitalized elderly patients that encompasses social support, comorbidities, functional dependence as opposed to simply identifying a patient in a dementia setting.

As demonstrated in other studies, dementia was also signiﬁcantly associated with low body mass index (BMI) and poorer nutritional status prior to admission.^24^

Limitations of present study are the convenience sample from a single service and the difference in type of hospitalized elderly patient, since the sample of patients was drawn from a specialized geriatric nursing ward. Elderly patients admitted in this setting usually have more comorbidities, greater frailty, use more medications, including psychotropic combinations, tend to have greater functional decline and longer hospital stays, increasing the risk of developing *delirium* and infections during hospitalization. Another limitation was the study design. Prospective studies can produce more valid results for investigations of demographic and clinical conditions associated with hospitalization of patients with dementia. The main contribution of the study is the identiﬁcation, in a developing Latin American country, of medical conditions associated with the hospitalization of elderly with dementia, possibly fostering the development of preventive and early management care to reduce the chances of subsequent hospital admission. From a strictly institutional standpoint, the early identiﬁcation and treatment of these conditions can reduce the average length of stay, risk of *delirium* , exposure to the hospital environment (e.g. risk of infections)^25^ and ultimately, the cost of hospitalization.

Another potential impact of our study pertains to the process of diagnosing dementia within the hospital environment. We note the need for better diagnostic instruments and improved awareness of certain medical conditions associated with dementia, which could lead to earlier diagnosis and its associated positive effects: early relief of symptoms, more effective management of medications and comorbid conditions that could otherwise worsen the patient's cognitive state, improved caregiver education about accident risks, and more timely ﬁnancial planning for the patient and family.^26^
